# Pilot–Scale Production of Carbon Hollow Fiber Membranes from Regenerated Cellulose Precursor-Part II: Carbonization Procedure

**DOI:** 10.3390/membranes8040097

**Published:** 2018-10-15

**Authors:** Shamim Haider, Jon Arvid Lie, Arne Lindbråthen, May-Britt Hägg

**Affiliations:** Department of Chemical Engineering, Norwegian University of Science and Technology (NTNU), 7491 Trondheim, Norway; haider@ntnu.no (S.H.); jonarvidlie@gmail.com (J.A.L.); arne.lindbrathen@ntnu.no (A.L.)

**Keywords:** molecular sieve, regenerated cellulose, carbonization process, gas separation

## Abstract

The simultaneous carbonization of thousands of fibers in a horizontal furnace may result in fused fibers if carbonization residuals (tars) are not removed fast enough. The optimized purge gas flow rate and a small degree angle in the furnace position may enhance the yield of high quality carbon fibers up to 97% by removing by-products. The production process for several thousand carbon fibers in a single batch is reported. The aim was developing a pilot-scale system to produce carbon membranes. Cellulose-acetate fibers were transformed into regenerated cellulose through a de-acetylation process and the fibers were carbonized in a horizontally oriented three-zone furnace. Quartz tubes and perforated stainless steel grids were used to carbonize up to 4000 (160 cm long) fibers in a single batch. The number of fused fibers could be significantly reduced by replacing the quartz tubes with perforated grids. It was further found that improved purge gas flow distribution in the furnace positioned at a 4-degree to 6-degree angle permitted residuals to flow downward into the tar collection chamber. In total, 390 spun-batches of fibers were carbonized. Each grid contained 2000–4000 individual fibers and these fibers comprised four to six spun-batches of vertically dried fibers. Gas permeation properties were investigated for the carbon fibers.

## 1. Introduction

Polymeric membrane-based gas separation process had already been proven commercially [1,2] when Koresh and Soffer [3,4,5,6] produced and reported the first carbon molecular sieve (CMS) membranes in the 1980s. Although polymeric membranes still dominate the market for gas separation due to a low production cost [1,7], the applications of these membranes are restricted by fairly low performance (meaning the inevitable trade-off between selectivity and permeability [7]) and usually poor chemical and thermal resistance. On the contrary, carbon membranes prepared from their polymeric precursor membrane have shown both excellent separation performance as well as thermal and chemical resistance when investigated in different applications [8,9,10,11,12].

Since CMS are a relatively new class of membranes, most of the reported research is done on a laboratory scale [10,13,14,15,16,17,18,19,20,21,22,23] and there are limited data available on commercial success [24,25]. The major challenges to commercially produce these membranes are that most of the precursors being used (polyimides, polyetherimide, phenolic resin etc. [26]) are relative expensive materials or are obtained only at a laboratory scale. They have low mechanical strength (especially shear strength) and high production cost [27]. The aim of this study was to develop a cheaper process to produce CMS membranes on a pilot-scale. Cellulose esters and in particular cellulose acetate (CA) have a relatively low cost and have been widely used in the membrane industry [28,29]. However, direct carbonization of CA results in discontinuous carbon (more like a powder) since the intermediate product levoglucosane [30] is not formed during carbonization. Hence, the CA must be deacetylated to form “regenerated cellulose” after the membrane casting process, e.g., the dry-wet spinning process. In 1995, Soffer et al. [31] patented the protocol for the carbonization of cellulose precursor. Carbon Membranes Ltd. (Arava, Israel) produced cellulose-based carbon hollow fiber modules on a pilot scale to successfully recover SF_6_ in dielectric environments, but the company was closed after a few years [24,32,33].

CMS membranes from regenerated cellulose precursor have shown excellent gas permeation properties on both the lab and pilot scale [13,14,18,25,34,35,36]. MemfoACT AS (Trondheim, Norway), which is a company that has closed down, produced regenerated cellulose-based carbon membranes on semi-commercial plant using a multi-step production method (spinning of CA fibers, solvent-exchange, deacetylation, solvent-rinsing, drying, carbonization, module construction, and gas permeation testing) [12].

Carbonization is a critical step in the process of forming carbon membranes and the resulting pore structure (which depends on the carbonization steps and morphology of precursor) will determine the separation properties of the resulting membrane. The gases and tars liberated upon carbonization depend on the chemical composition of the precursor and can include CO_2_, CO, H_2_, N_2_, NOx, and H_2_O. A carbon residue is also formed by condensation of polynuclear aromatic compounds and expulsion of side chain groups [37]. If these by-products are not removed fast enough, they can deposit it in the carbon matrix, which makes it denser (very low flux) and, in the case of thousands of fibers, they may fuse together [38].

Generally, the carbonization furnace is used in horizontal orientation with one or three heating zones and a quartz tube reaction chamber. The loading of thousands of hollow fibers in a horizontal quartz tube would result in fused fibers (unusable) because of uneven distribution of purge gas flow (described below) in the tube and then residual products staying under the fibers makes them stick together. 

To overcome the problem of fused fibers, Karvan et al. [39] tested a pilot-scale system with a vertically oriented furnace to carbonize the precursor hollow fibers. They carbonized 242 fibers using a “loop system” to suspend the fibers individually in the vertical position to reduce the possibility of fibers contacting during carbonization and to promote the flow of purge gas and by products. The maximum survival rate of fibers was 93% and the rest of the fibers were broken on the top. The fiber broke due to softening of the material, which transitioned from glassy to the rubbery state. These fibers had 66% lower permeance of CO_2_ and 16% decreased CO_2_/CH_4_ selectivity when compared to their laboratory/bench scale carbon hollow fibers.

The production process for several thousand carbon fibers in a single batch is being reported for the first time ever. MemfoACT AS carbonized regenerated hollow fibers in a 3-zone horizontal furnace. They used different types of perforated stainless-steel flat trays to carbonize up to 4000 (160 cm long) fibers in a single batch with maximum survival of 95%. Fiber shrinkage during carbonization was 30% to 35%. The permeation properties of carbonized fibers were almost similar or even higher in some batches when compared to the lab-scale process. In total, 390 spun batches of fibers were carbonized using quartz tubes and perforated plates to investigate and enhance the survival rate of carbonized fibers. The purge gas flow distribution in all directions of the fibers was clearly improved by using perforated plates. Hence, the aim of the research by MemfoACT AS was to use a relatively inexpensive, environmentally-friendly membrane material (CA) to make carbon membranes at a semi-industrial scale with maximum successful fibers and acceptable gas separation performance. However, the focus of this paper is more on the carbonization process that was used by MemfoACT AS and briefly describes the precursor preparation. The performance of produced CM and the survival rate of each batch is also reported. Predicting the cost of carbon membrane modules is difficult because of the lack of commercial precedent. Polymeric hollow-fiber membranes are assumed to cost roughly $20–50/m^2^. The cost of carbon membranes is far more uncertain. It is expected that carbon membranes cost between one-order and three-orders of magnitude more per unit of the membrane area compared to polymeric membranes [1,40,41]. This would make the range a rather daunting $200–50,000/m^2^. However, the goal of MemfoACT was to produce regenerated cellulose-based carbon membranes in the range of $100–200/m^2^. The details about the production cost and techno-economic feasibility of CM-based on a pilot scale production price has been discussed in our previously reported work [25,27,42].

## 2. Experimental

### 2.1. Preparation of Precursor Fibers

**Figure 1:**
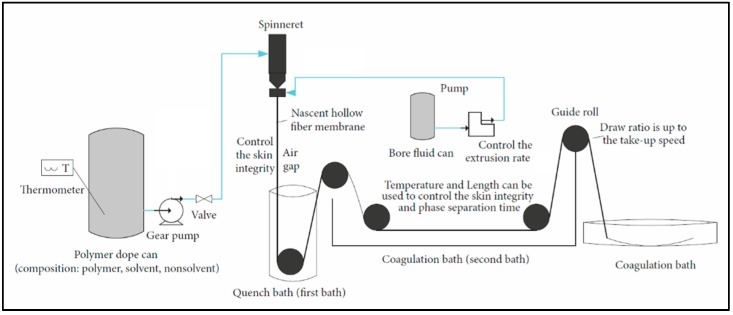
Schematic diagram of the hollow fiber spinning process.

A dope solution consisting of CA, *N*-methylpyrrolidone (NMP), and polyvinylpyrrolidone (PVP, M_W_ 10,000 from Sigma Aldrich, Oslo, Norway) (CA 22.5%/NMP 72.5%/PVP 5%, all *w*/*w*) was prepared. A bore solution consisting of water/NMP in different ratios was used as an internal coagulant while water at 50 °C was used as an external coagulant for the spun fibers. A well-known dry/wet spinning process was used on a pilot scale spinning set up, delivered by Philos Korea, to spin the CA hollow fibers. Each batch consisted of 2 m long 1200 fibers. The fibers were treated with aqueous solution of glycerol for 24 h to completely wash out the bore solvent from the fibers and to preserve the porosity of the fibers. To deacetylate CA fibers, they were then immersed for 2.5 h in a 90 vol % 0.075 M NaOH aqueous solution diluted with 10 vol % Isopropanol. The regenerated cellulose fibers were treated with 7.5% glucose solution (aqueous) for 30 min to reduce the shrinkage and curliness of fibers after the drying process. The number of days before loading the furnace for the carbonization process varied from 1 to 10. Figure 1 shows the schematic diagram of the hollow fiber spinning process.

### 2.2. Fiber Loading in the Furnace and Carbonization Procedure

#### 2.2.1. Description of the Furnace

**Figure 2:**
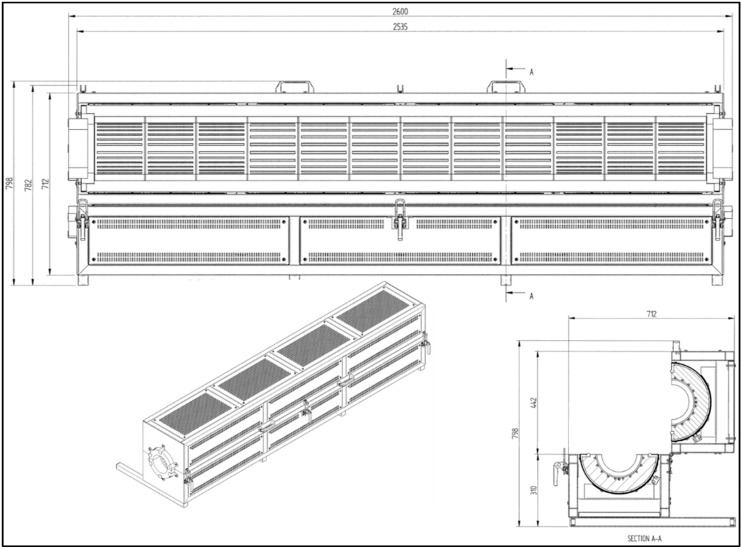
Drawing of 3-zone furnace with dimensions (mm) (Source: Carbolite).

**Figure 3:**
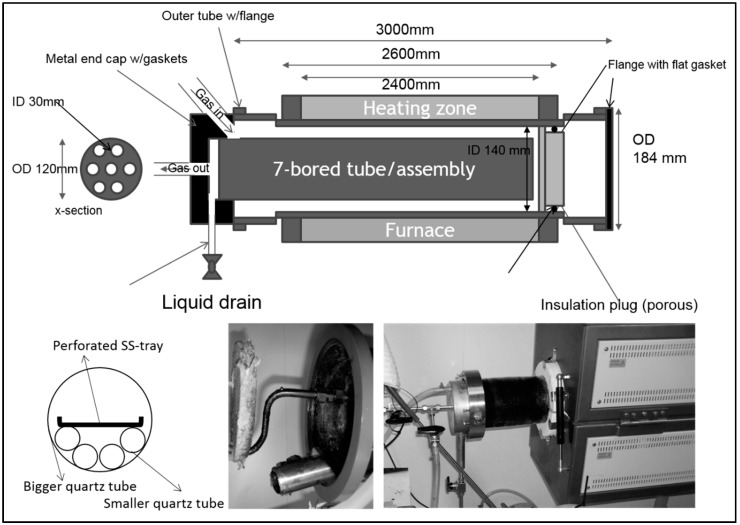
Drawing of furnace, bigger quartz tube, smaller quartz tube assembly and photographs of the system with carbonization in progress.

The furnace used in this work was specifically made (Model: Carbolite special HZS 12/150/2400, bought from VWR International AS, Hope Valley, England) with three independently controlled heating zones. A custom-built stand under the furnace was used to adjust the height accordingly. The height of the furnace could be adjusted independently on each end. The drawing of the furnace is shown in Figure 2. A quartz tube with outer diameter (OD) of 150 mm, 5 mm wall thickness, and 3000 mm in length and seven smaller quartz tubes with 34 mm OD, 2mm wall thickness, and 2700 mm in length were purchased from Chemi-Teknik AS, Oslo, Norway. One end of the bigger tube was sealed with gasket and stainless-steel flange. However, the other end was used for loading/unloading of the small quartz tubes and gas inlet/outlet connections. Glass wool (can withstand 800 °C) was used for an insulation purpose and was provided by Rockwool Colnite AS, Oslo, Norway. Gaskets and stainless-steel clamps as well as cup/flanges were custom-built in the workshop of Norwegian university of Science and Technology. A complete drawing of the furnace system is presented in Figure 3 along with photographs of the furnace. Gas flow controllers were bought from Aalborg USA and gases were delivered by Yara Praxair AS, Trondheim, Norway.

Two types of 2 m long perforated plates (purchased from Nisjemetall AS, Røyken, Norway) with square openings (10 × 10 mm^2^ and 20 × 20 mm^2^) and width of 120 mm were also used to carbonize the hollow fibers.

#### 2.2.2. Procedure for Carbonization

Different types of collection methods were tried. However, only two methods that had a great effect on membrane morphology after deacetylation, drying, and carbonization are being reported here.

##### Loading the Furnace

The angle between support/level and furnace/tube was set to 6° by raising the closed end of the furnace to enhance the flow of residue downward. 1–7 quartz tubes were filled with 500 to 1000 fibers in each tube. A thread with a loose knot around the fibers was used to pull the fibers inside the quartz tubes. The thread was removed after fibers are inserted. An insulation plate was cut OD 120 mm and holes were made according to the template (honey comb arrangement), which is shown in Figure 3 (number of tubes loaded, 30 mm hole diameter), a 32 mm OD hole for the tar drain tube, and a 3/8″ OD sweep gas tube. Insulation plate along with quartz tubes were pushed inside the bigger/furnace tuber into the heating zone of the furnace (46–48 cm from the edge of the furnace tube).

In the case of using the perforated plate/grid, the grid was filled with 1600 to 4000 fibers. The fibers were distributed as equally as possible on the grid with the layer thickness across the width of the grid becoming as constant as possible. The upper end of the fiber (the upper end during the drying of cellulose fibers) was placed at an upstream end of the grid (the end of the furnace tube where preheated purge gas enters). Flat grids were unstable when placed directly into the furnace tube. Therefore, four smaller quartz tubes were used as support inside the furnace tube in the bottom of grids (also shown in Figure 3). It also helped to protect the inner wall of the furnace tube from scratches (stainless-steel grids) and provided distance between purge gas and the grid. The grid was then pushed into the heating zone of the furnace (46–48 cm from the edge of the furnace tube).

**Figure 4:**
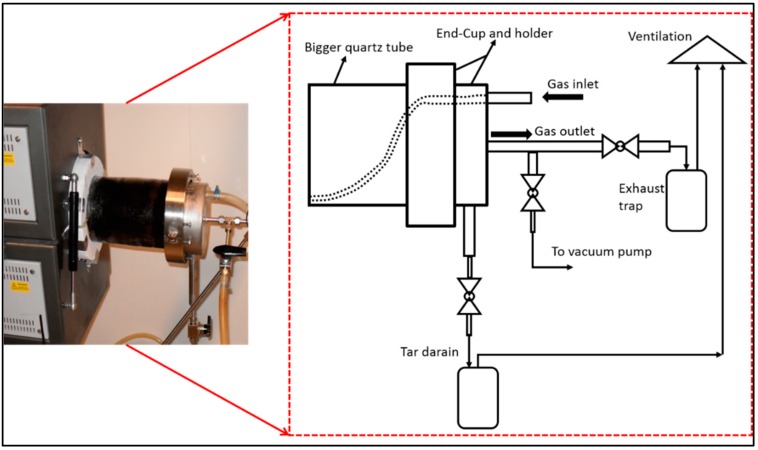
A photograph (on **left**) and a schematic diagram (on **right**) of end-cup and holder on the fiber loading-end of the furnace tube.

**Figure 5:**
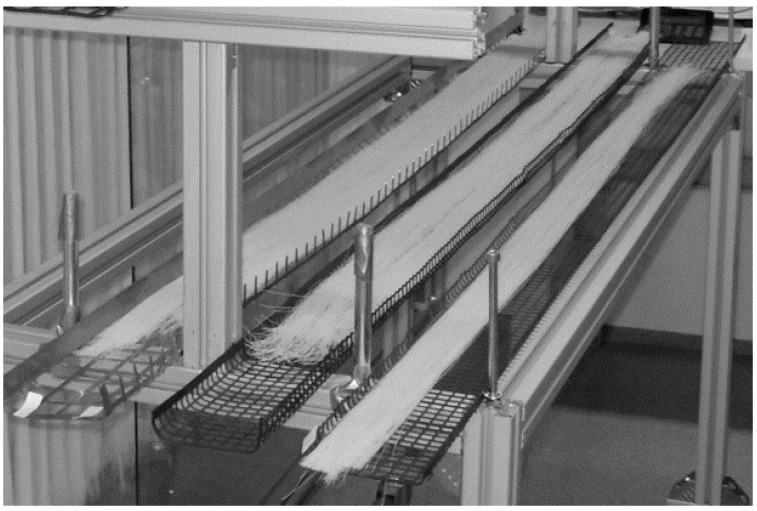
Perforated plates with square openings (10 × 10 mm^2^) and (20 × 20 mm^2^).

The inlet of the purge gas (flowing into the furnace) tube was placed in between the middle support tubes and the outlet end was pushed through the insulation plate (demonstrated in Figure 4). Then the tar drain tube was inserted into the insulation plate, which makes an angle so that the tar flows to the end cup/flange of the furnace. This is shown in Figure 3. The flange, gasket, and metal cup were greased with Loctite 8104. The purge gas inlet tube is by design welded to the cup and coupled with a tube inside the furnace through a tight silicon tube (ca 40 mm long), as illustrated in Figure 4. Two clamp halves were mounted, holding the cup, with two screws so that both halves gently touched each other. The screws in the clamps were tightened very gently, avoiding high momentum, to protect the quartz tube. Figure 5 shows the photograph of the perforated plates loaded with fibers, which are ready for carbonization.

All the valves separating the furnace from the rest of the gas lines were closed. Then the vacuum pump was turned on followed by slow opening of the valve between the pump and the furnace. Then the system was checked for any possible leakage. The system was left to evacuate overnight, which normally yields a pressure <10 mbar.

##### Carbonization

After evacuating the air out of the system, N_2_ or CO_2_ flow of 0.8 L/min was supplied through a gas flow controller. The flow was gradually increased to 1.9 L/min to fill the oven. As soon as the pressure inside the furnace tube reached just above the atmospheric pressure (1.1–1.2 atm), the valve to the exhaust trap and the valve to the tar trap was slowly opened. H_2_O/NMP in the volume ratio of 4/1 was used as an exhaust trap on the outlet of the furnace. For tar absorption, 10% triethyleglycol (TEG) in water was used and an outlet from both the exhaust trap and the tar trap was then connected to the ventilation. Gas flow was varied for a few batches to optimize, according to the fiber holders (no. of quartz tubes or SS-grid), but it was always kept in the superficial velocity range of 1 to 10 cm/min [38].

**Figure 6:**
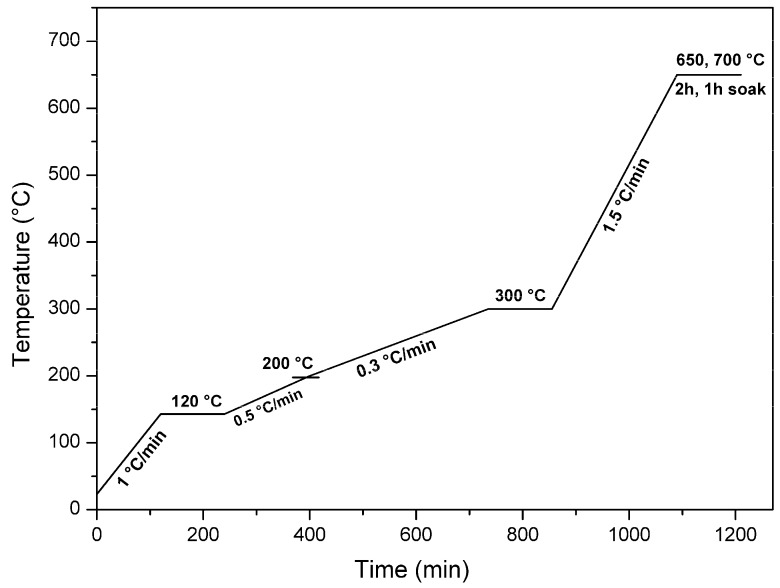
Carbonization protocol used by MemfoACT AS.

The carbonization protocol (as illustrated in Figure 6) was then started. At the end of the protocol, the system was left to cool naturally and gas continued to flow at the original flow rate. When the furnace temperature was 70 °C or below, the gas flow was stopped and both traps (exhaust and tar) were disconnected. Because the cooling of the furnace may create a slight vacuum inside, the liquid in the exhaust trap is thus hindered to flow into the furnace tubes or tar trap. Quartz tubes/grids were pulled out of the furnace tube and fibers were stored on a clean surface (a plain paper). Then fibers were left to degas overnight before they were further processed. The stainless-steel grid did not need any washing after carbonization since there was no residue stuck on the grid. However, the quartz tube needed washing after each carbonization cycle.

### 2.3. Permeation Testing

For the permeation experiments discussed here, CM (0.002–2 m^2^ for each module) modules were tested in a permeation set-up with a shell side feed configuration. The mass transport properties of CM were measured with the single pure gases CO_2_, N_2_, CH_4_, and mixed gas (40% CO_2_ in CH_4_) at 5 bar feed pressure (ambient temperature: 20–23 °C) and with or without vacuum on the permeate side. He et al. [14] has also performed the mixed gas experiments on the carbon membrane (prepared with a similar protocol) and results showed that the membrane performance for CO_2_ separation is the same or even higher in some cases for mixed gas as compared to single gas separation. The carbon membranes prepared by MemfoACT AS also showed similar results. The values for CH_4_ gas were found to give a selectivity α CO_2_/CH_4_ = 3·α CO_2_/N_2_.

The performance of the membrane was evaluated by measuring the CO_2_ permeance in [m^3^(STP)/(m^2^ h bar)] and CO_2_/N_2_ selectivities (α) by using Equations (1) and (2). The tests were run from several hours to several days to ensure that the transient phase of diffusion was passed and the steady state obtained (*dp*/*dt* tends to a constant). The gas permeance, *P* [m^3^(STP)/(m^2^·h·bar)], was evaluated by using Equation (1).

(1) $$P=\frac{9.824\;V\cdot (dp/dt)}{\Delta P\cdot A\cdot T_{exp}}$$

In this case, *V* is the permeate side volume (cm^3^) that can be measured with a pre-calibrated permeation cell reported elsewhere [15,40]. However, the permeate side volume for this study was estimated by the tube length and the cylinder volume on the permeate side. *dp*/*dt* and *A* are the collection volume pressure increase rate (m bar/s) and the total active area of the membrane (cm^2^), respectively. Δ*P* (bar) is the pressure head and *T_exp_* (K) is the temperature for the experiment. The ideal selectivity was defined as the ratio of the pure gas permeance, which is shown in Equation (2).

(2) $$\propto_{A/B}=\frac{P_{A}}{P_{B}}$$

## 3. Results and Discussion

### 3.1. Fiber Morphology and Successful Fibers

**Figure 7:**
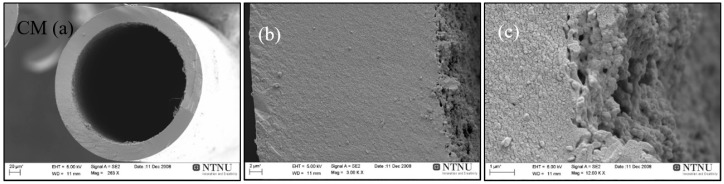
SEM images of carbon hollow fiber membrane (CM), (**a**) cross section, (**b**) wall magnified, and (**c**) inner edge of the wall magnified.

Figure 7a–c shows the scanning electron microscopic (SEM, Zeiss SUPRA 55VP, NTNU) images of the carbon hollow fiber membrane. The average diameter of carbon fibers was 210 µm with wall thickness of 23 µm. Carbonization is a critical step and varying carbonization conditions would result in a dissimilar carbon matrix for each carbonized batch of hollow fibers. 

The final carbon fibers should not be perfectly straight but should preferably have waves (i.e., wavelength >10 cm) to (i) improve the gas flow pattern in the module and to (ii) handle thermal expansion or shrinkage without breakage. The color of carbonized fibers gave the first insight to evaluate if the fibers were processed as required. The carbonized bundle should be shiny, which means that surface of carbon fibers is free from low-molecular products (tars) of cellulose pyrolysis. The residual tars caused the fibers to stick together, which resulted in their embrittlement. To make a qualitative test on the carbonized bundle of fibers, some quick mechanical examinations were performed. The optimal bundle of carbon fibers should easily separate into individual carbon fibers with a gentle hand. The fiber bundle was shaken in a careful and gentle way by holding on one end, so any broken fibers could fall out. The procedure was repeated by holding the other end of the bundle the same way. Carbonization was considered successful if the broken fibers do not exceed 2% of the total fibers in the bundle. Fiber strength was tested by a simple loop diameter method. A loop made of an individual carbon fiber was slowly tightened while measuring the diameter simultaneously until the fiber was broken. An acceptable average minimal diameter should not exceed 10 mm. Sampling was performed on different parts of the fibers as well as from the middle and outer part of the bundle to evaluate the uniformity of its mechanical properties.

**Figure 8:**
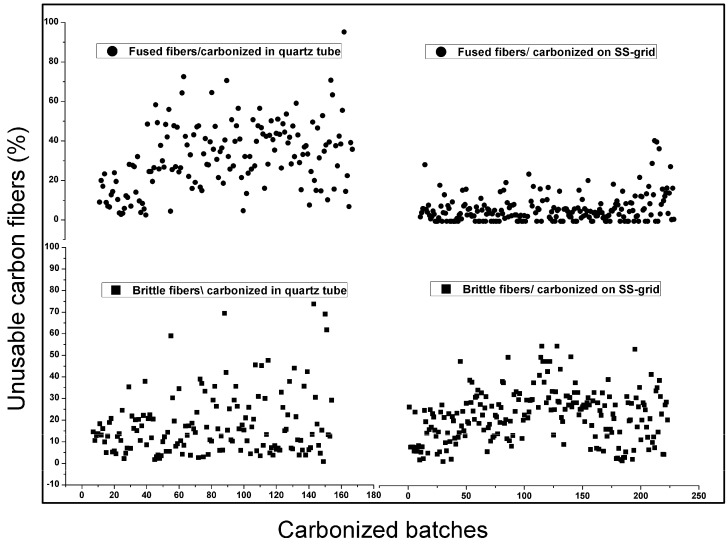
Fused and brittle fibers after carbonization, results with quartz tube (**left**), and results with SS-grid (**right**).

Figure 8 summarizes the results of 390 spun-batches, which are carbonized both in the quartz tubes and on the SS-grid. As can be seen in Figure 8, on average, 40% of the total carbonized fibers and fibers in the quartz tubes were fused and unusable. However, some batches exceeded to 60% of fused fibers. The brittle fibers obtained from the same batches were in the range from 0% to 30%. The percentage of curly and collapsed fibers (not shown here) was between 0% and 5%. Hence, the “survival rate” of these batches was very low (<10%). It was assumed (i.e., % of good fibers) that honey comb arrangement of quartz tubes might create more uniform conditions in cross-section within each bundle, but it was observed that the unequal flow rate of gas was distributed in each bundle/tube. Therefore, most of the residual produced during carbonization stayed inside the bundle, which resulted in fused and brittle fibers. Using an angle on furnace (4–6° by raising the closed end of the furnace to enhance the flow of residue downward) improved the rate of survival but still could not produce successful fibers consistently. The residual amount was also different in all batches depending on the changed parameters during precursor preparation or the number of fibers in each carbonization batch. It could be noted that the survived fibers were always on the top of the bundle and those in the bottom part was fused.

The number of fused fibers were significantly reduced when carbonized on the perforated SS-grids, which is shown in Figure 8. Usually each perforated SS-grid contained 2000–4000 individual fibers and these fibers included 4–6 spun-batches of vertically dried regenerated cellulose fibers. Initially, all fibers on the grid were placed in the same direction (top side during drying) for all batches. After several carbonizations, it was observed that the fibers fused more only on one side of the bundle (top side). Hence, the dried batches were arranged on an SS-grid in an alternating order (top of one batch in one direction neighboring with the bottom of the other batch and so on). It improved the number of survival fibers but there were still some fused fibers in each bundle. Then a perforated grid with bigger openings (20 × 20 mm^2^) was used, which increased, and the results were almost similar as with previous grid (10 × 10 mm^2^). It was found that fibers on the bottom of the bundle touching to the grid and specifically sections of the fibers in contact with SS-grid were sometimes fused and got stuck with the grid. That portion of the fibers became brittle and pulling it away from the bundle (separating the fibers) would break the fibers. Although numerous batches had zero fused fibers, it was still challenging to keep the consistent production rate. While gas distribution was improved by the use of grids, there were still some sections where the pressure drop was higher (fibers not equally dispersed on grid) and gas was not able to isolate the fiber-tar-fiber and fiber-tar-grid connections. 

The carbonized hollow fibers should be sufficiently strong, flexible, and uniform to produce bigger commercially modules with high packing density. The challenge during carbonization is the fiber brittleness. As shown in Figure 8, both carbonization methods (fibers inside quartz tube and on SS-grid) had almost a similar number of brittle fibers. These fibers could not be looped into the 10 mm diameter before they broke. This was used as the definition of brittleness in this study. There may be two possible reasons for the brittle carbon fibers: (1) varying properties of the precursor in each batch, e.g., partial deacetylation, fast drying at lower relative humidity (40–30%), etc. (2) surface of carbon is not fully free from low-molecular products (tar). In future research, a continuously rotating perforated tube (SS or glass) is suggested for the carbonization of big batches. This would help distribute the gas equally in a more efficient way, remove the residual tar, and avoid the continuous contact of fibers with tube and each other. Furthermore, a model to estimate the gas flow pattern inside the carbonization chamber would be very helpful to manipulate the gas distribution inside the chamber for a homogenous flow.

### 3.2. Gas Permeation Properties

The separation properties of the resulting membrane will be determined by the pore structure formed during carbonization. It was observed that a high temperature (700 °C) carbonization resulted in a dense membrane with decreased CO_2_ permeability (up to 20 Barrer) and high CO_2_/CH_4_ selectivity (above 200). Yet, 650 °C final temperature improved the permeation properties of the resulting carbon membranes by sacrificing some of the selectivity.

**Figure 9:**
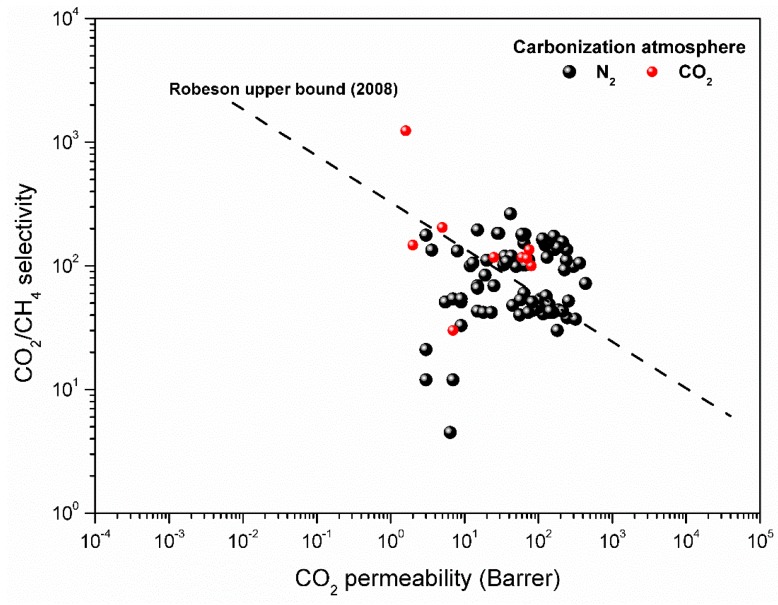
Separation performance of carbon hollow fiber membrane when carbonized under N_2_ and CO_2_ atmosphere (Final temperature: 650 °C) (1 Barrer = 2.736 × 10^−9^ m^3^(STP) m/m^2^ bar h).

Figure 9 presents the permeation results of the carbon hollow fiber membrane prepared from regenerated cellulose hollow fibers. As shown in Figure 9, some batches were carbonized under the CO_2_ atmosphere. Despite good permeation properties, the resulting fibers possessed very weak mechanical properties. Fibers carbonized under vacuum had lower CO_2_ permeability and selectivity values than when prepared in CO_2_ or N_2_ atmosphere. The membranes prepared in the N_2_ atmosphere exhibited high performance (as shown in Figure 9) and good mechanical properties. Therefore, the rest of the batches were exposed to the N_2_ atmosphere during carbonization. Geiszler and Koros [38] reported that an inert gas atmosphere resulted in a more open but a less selective CMS matrix compared to vacuum carbonization. They explained this with acceleration in the carbonization process due to an increased gas phase heat and mass transfer. The same authors also observed that the CO_2_ purge produced a highly porous, nonselective membrane by oxidizing the carbon and ten times reduction in purge gas flow rate caused a decrease in the permeate flux, which was presumable by the deposition of tar (carbon) either on the membrane surface or in the pores. Based on our own observations where carbonization in the CO_2_ atmosphere with previously mentioned flow rates looked promising, reasonable permeability and selectivity was achieved. The yield of produced carbon fibers was significantly reduced due to weak mechanical properties.

As already stated, the N_2_ atmosphere was chosen to be used for the rest of the batches. The optimized flow rate of purge gas yielded mechanically strong fibers with acceptable gas permeation properties. CM produced on a pilot-scale plant showed equal or higher performance as compared to the laboratory scale carbon membranes in CO_2_-CH_4_ separation. The performance of produced fibers is comparable with the previously reported work [13,14,15,17]. The results presented in Figure 9 are for the smaller (~0.002 m^2^ of each module) modules. However, the bigger modules with an effective area of 2–2.5 m^2^ of each module were made and used to produce vehicle fuel from biogas. The performance of these bigger modules was enhanced further by a chemical vapor deposition (CVD) process. Details about the construction of bigger modules, performance, CVD procedure, and aging of these modules were reported elsewhere [25]. Preparation of regenerated cellulose-based carbon membranes, characterization, and the performance of these modules on a laboratory scale has already been reported [14]. The carbonization process is not stable enough to achieve the same results for each fiber in each batch. However, the idea of this work was to keep the variance minimal within and between batches. It is important to note that the results shown in Figure 9 were obtained by testing several fibers from different batches. The performance of different fibers picked up from different batches varies. This varying performance may be due to a change in the structure that might have occurred during the preparation of precursor fibers or through the carbonization process. In each bundle of carbonized fibers, there might be a gradient of tar build up. In some cases where membrane performance is below the upper bound, it seems that a smaller number of open pores are available for gas permeation. During carbonization, it might be that tar have stuck the fiber surface to reduce the number of open pores, which ultimately declined the performance of the membrane. However, in other batches, the chosen fiber for permeation testing might have had more open pores.

Karvan et al. [39] reported that fibers carbonized on a pilot scale plant in a vertically-oriented furnace had 66% lower permeance of CO_2_ and 16% decreased CO_2_/CH_4_ selectivity when compared to their laboratory/bench scale carbon hollow fibers. The gas permeation results of carbon membranes prepared by MemfoACT AS showed that horizontal orientation could give carbon membranes with a high performance. An adjustment of the furnace angle (4–6° by raising the closed end of the furnace) and a perforated plate inside the furnace increased the number of good fibers consistently by securing equal purge gas distribution and removing the residual products simultaneously.

## 4. Conclusions

A pilot-scale system for production of high performance carbon membranes from regenerated cellulose has been studied and reported. A relatively low-cost precursor material and a single stage carbonization process, which obtained over 90% of successful carbon fibers, made the pilot production of carbon membranes more economically attractive. The number of fused fibers was significantly reduced by replacing the quartz tubes as fiber carriers inside the furnace with perforated trays of stainless steel as fiber carriers. Separation performance of the regenerated cellulose-based carbon membranes was equal or even higher than the reported membranes produced on a laboratory scale. Fibers carbonized under a CO_2_ atmosphere had lower mechanical properties compared to the fibers carbonized under an N_2_ atmosphere. The carbonization process is not stable enough to achieve the same results for each fiber in each batch. However, the idea of this work was to keep the variance minimal within and between batches. A few suggestions were proposed to enhance the survival rate of good fibers with more consistency. Through careful optimization of the carbonization process (temperature, purge gas flow rate, purge gas type, and angle of the furnace), the number of fused fibers may be reduced. An adjustment of the furnace angle (4–6° by raising the closed end of the furnace) and a perforated rotating tube inside the furnace may increase the number of survived fibers consistently by securing equal purge gas distribution and removing the residual products simultaneously. Exploration of the purge gas flow pattern inside the furnace is also proposed in future work.
